# Parasitism, sexual dimorphism and effect of host size on *Apocephalus attophilus* offspring, a parasitoid of the leaf-cutting ant *Atta bisphaerica*

**DOI:** 10.1371/journal.pone.0208253

**Published:** 2018-12-03

**Authors:** Cliver Fernandes Farder-Gomes, Verônica Priscila da Silva, Thalles Platiny Lavinscky Pereira, José Eduardo Serrão, Evaldo Martins Pires, Marco Antonio Oliveira

**Affiliations:** 1 Departament of Entomology, Federal University of Viçosa, Viçosa, Minas Gerais, Brazil; 2 Institute of Biological Sciences, Federal University of Viçosa *Campus* Florestal, Florestal, Minas Gerais, Brazil; 3 Departament of Zoology, University of São Paulo, São Paulo, São Paulo, Brazil; 4 Postgraduate Program in Environmental Sciences, Federal University of Mato Grosso C*ampus* Sinop, Sinop, Mato Grosso, Brazil; Arizona State University, UNITED STATES

## Abstract

*Atta bisphaerica* (Forel) is a leaf-cutting ant that specializes on grass and causes productivity losses in sugar cane fields and pastures. Three phorid species, *Apocephalus attophilus* (Borgmeier), *Myrmosicarius grandicornis* (Borgmeier) and *Eibesfeldtphora bragancai* (Brown), have been found parasitizing *A*. *bisphaerica* workers. These parasitoids can reduce plant material transported into the nests and ant traffic on the trails. Therefore, phorid flies have been considered potential biological control agents for leaf-cutting ants. Here, we evaluated which parasitoid species attack the leaf-cutting ant *A*. *bisphaerica* in pasture areas of a Brazilian Savannah-Atlantic Forest ecotone, parasitism rate, effect of host size, sexual dimorphism and sex ratio of the emerged parasitoids. Four nests of *A*. *bisphaerica* were selected in pasture areas from August 2016 to August 2017, with 400 workers collected from each colony monthly. A total of 23,714 *A*. *bisphaerica* workers were collected during the study, of which 236 (0.99%) were parasitized by phorid parasitoids. *Apocephalus attophilus*, *E*. *bragancai* and *M*. *grandicornis* parasitized 217, 17 and 2 ants, respectively. The higher parasitism rate was found in the hottest/rainy season of the year. Non-parasitized ants survived longer than those parasitized by *A*. *attophilus*. The larval and pupal periods of this parasitoid were 2.2 ± 0.8 and 16 ± 1.4 days, respectively, and the number of pupae per parasitized ant ranged from 1 to 7. The number of *A*. *attophilus* pupae per host increased with the host head size. Likewise, the size of the adult parasitoids also increased according to the host ant. *Apocephalus attophilus* females were larger than males and the sex ratio (male: female) did not differ from 1: 1. Our results showed that *A*. *attophilus* would be a potential biocontrol agent of leaf-cutting ants because it produces multiple larvae per host, allowing a great production of parasitoids with short developmental time and kills the host ant faster than other phorids.

## Introduction

Leaf-cutting ants of the genus *Atta* and *Acromyrmex* (Hymenoptera: Attini) are recognized as important agricultural and forest pests, cutting fresh plant material from a wide range of plant species for cultivation of the symbiont fungi, which feed the entire colony [1,2]. *Atta bisphaerica* (Forel) is a grass-cutting specialist that causes losses in sugar cane and managed pastures [3,4]. The loss in annual sugar cane productivity in Brazil caused by these insects may exceed three tons per hectare [4]. Insecticides, mainly granular toxic baits, are the most common method to control leaf-cutting ants [1], but pesticides represent a significant risk for humans, non-target organisms and the environment [5–7]. Use of biological agents for pest suppression is a promising area, but development of biological control programs requires knowledge of the biology and behavior of natural enemies [8,9].

Phorid flies (Diptera: Phoridae) have been the focus of research as biological control agents [10–12]. Success obtained in the control of fire ants *Solenopsis* spp. with phorid parasitoids of the genus *Pseudacteon* spp. [13–15] reinforces the possibility of using these natural enemies to manage leaf-cutting ants. The presence of phorids changes ant behavior, reduces ant traffic on trails and the amount and weight of plant material transported [11,12,16].

Three phorid species, *Apocephalus attophilus* (Borgmeier), *Myrmosicarius grandicornis* (Borgmeier) and *Eibesfeldtphora bragancai* (Brown), have been found parasitizing *A*. *bisphaerica* workers [17,18], with a parasitism rate of 4,37% [18]. In addition, parasitism rate by these three species increases with the decreasing temperature, and *E*. *bragancai* exhibited higher parasitism in high relative humidity, while *A*. *attophilus* exhibited in dry seasons [18]. The number of *A*. *attophilus* offspring increases with *A*. *bisphaerica* head size and the number of pupae per host can reach 1 to 10 [18]. Likewise, the size of the *E*. *bragancai* and *M*. *grandicornis* adults increase with host ant size [18,19], but there is no data for *A*. *attophilus*.

Phorids that parasitize leaf-cutting ants can coexist in the same site, attacking ants of different head sizes [8,20]. In the case of *A*. *bisphaerica*, *E*. *bragancai* develops in the largest workers, whereas *A*. *attophilus* and *M*. *grandicornis* develop in the smallest ones [18]. Thus, the presence of multiple phorid species, developing on different host sizes, may provide more effective control of leaf-cutting ants [8,18].

*Apocephalus attophilus* is a parasitoid of the leaf-cutting ants *A*. *bisphaerica*, *Atta sexdens* (Linnaeus), *Atta laevigata* (Smith) and *Atta cephalotes* (Linnaeus) [21–23]. Females attack leaf carrier workers, depositing an egg in the head of the ant, with their larvae feeding on the cephalic content, killing the ant and pupating out of the host body [21,23].

In phorid flies, sex ratio (male: female) vary according to host size or temperature [9,24–26] and females are commonly larger than males [27,28]. Ant body size is found to be correlated with fecundity, longevity, host-finding ability and mating success of parasitoids [24,29,30].

Here, we report some biological and ecological characteristics of the interaction between the leaf-cutting ant *A*. *bisphaerica* and phorid parasitoids in pasture areas of a Brazilian Savannah-Atlantic Forest ecotone. Specifically, the aim of this study was to investigate: (1) Which parasitoid species attack *A*. *bisphaerica*, (2) parasitism rate, (3) the relationship between host size and number and size of phorid offspring, (4) sexual dimorphism and (5) sex ratio of the emerged parasitoids.

## Material and methods

### Study site and collection of ants

The study was carried out at Universidade Federal de Viçosa—*Campus* Florestal, municipality of Florestal, Minas Gerais, Brazil, a Savannah-Atlantic Forest ecotone. The pasture areas were close to well-preserved florest fragments and none of the nests have undergone any form of chemical control.

Four different *A*. *bisphaerica* nests were selected in pasture areas (N1: 19° 52’ 48,303” S and 44° 24’ 49,112” W; N2: 19° 53’ 4,841” S and 44° 24’ 36,512” W; N3: 19° 53’ 4,704” S and 44° 24’ 35,994” W; N4: 19° 52’ 58,513” S and 44° 24’ 43,927” W), from August 2016 to August 2017, with 400 workers collected from each colony monthly between 07:30 AM and 11:00 AM in the foraging trails. When the ants were not foraging, a stick of wood was introduced into the nest entrance and as soon as the ants left the nest, they were collected.

### Rearing insects

In the laboratory, ants of the same nest were kept in a common plastic tray (28.4 × 18.8 × 6.1 cm) and fed daily with 10% honey water solution. These trays were stored at a temperature of 25 ± 1°C, relative humidity of 80 ± 5% and photoperiod of 12:12 h (light: dark) in a growth chamber. We checked all the trays daily, looking for new dead ants, puparia and adult parasitoids. Ants that died were removed daily, transferred to an individual glass tube (20 × 200 mm) and maintained in the same chamber. After three days, dead ants were observed under stereomicroscope to evaluate if they were parasitized by phorids [31]. Dead ants that had been parasitized could be visually determined by the presence of parasitoid pupae between the jaws, inside the head or outside the host [17,19,31]. Ants that were still alive 15 days after collection were discarded because they had not been parasitized [31].

Parasitized ants were kept in the growth chamber until the emergence of adult parasitoids, which were stored in vials containing 70% ethanol. The species and sex of these adult parasitoids were identified with the aid of descriptions and identification keys [22,32–34]. Voucher specimens were deposited at the Laboratory of Entomology/ Myrmecology UFV—*campus* Florestal.

Survival curves of non-parasitized and parasitized ants were plotted as percentage survival verses time. Non-parasitized ants were selected at random from the pool of collected ants. For ants, head width was measured as the width of the head capsule across the lower margin of the compound eyes and used as an indicator of parasitized ant size. Mesonotum width at its widest point of phorid males and females was used as an indicator of phorid size for comparisons between the size of parasitized ant and emerging male and female flies [18,35]. Wing length of phorids was measured as the length of the outstretched wing from the base to the apex and used as an indicator of parasitoid size for comparison between males and females [35]. The larval period of the phorids were estimated as the difference in days between the date of ant collection and pupae formation. The ants collected were already parasitized and we did not know the real oviposition date, thus, larval period was underestimated. The pupal period was the difference in days between the date of pupae formation and adult emergence. All measures were obtained with a millimeter ocular lens coupled to stereomicroscope.

### Data analysis

Student’s t-test was performed to compare temperature and precipitation in the hottest and rainy season from September to February and in the coldest and drier season from March to August. Chi-square test was performed to compare parasitism rate between these two periods. Kaplan-Meier estimator was used to generate survival curves of non-parasitized and parasitized ants. The log-rank test was used to compare the survival curves. Pearson’s correlation analysis was performed between the number of *A*. *attophilus* emerged per host and size of the parasitized ant. Another Pearson's correlation was performed to test correlation between host ant size and size of emerging parasitoids. Student’s t-test was performed to assess the differences in the wing length of parasitoid males and females. Lastly, the sex ratio of the parasitoids was statistically compared using chi-square test. All analyses were performed using GraphPad Prism version 7.0 for Windows (GraphPad Software, La Jolla, California, USA).

## Results

A total of 23,714 *A*. *bisphaerica* workers were collected during the study, of which 236 (0.99%) were found to be parasitized by phorid parasitoids. *Apocephalus attophilus* (Borgmeier), *Eibesfeldtphora bragancai* (Brown) and *Myrmosicarius grandicornis* (Borgmeier) parasitized 217, 17 and 2 ants, respectively.

The highest parasitism was found in November (88 parasitized ants) and the lowest in March (3 parasitized ants). Temperature (*t* = 4.151; *P* = 0.002) and precipitation (*t* = 5.063; *P*<0.0001) were higher in the hottest/rainy season (22.6 ± 0.2°C and 195.8 ± 31.1 mm, respectively) in relation to the coldest/drier (18.5 ± 0.1°C and 27.8 ± 11.3 mm, respectively). Parasitism was higher (χ^2^ = 37.441; *P*<0.0001) in the hottest/rainy season (165 parasitized ants) than in coldest/drier (71 parasitized ants).

**Fig 1:**
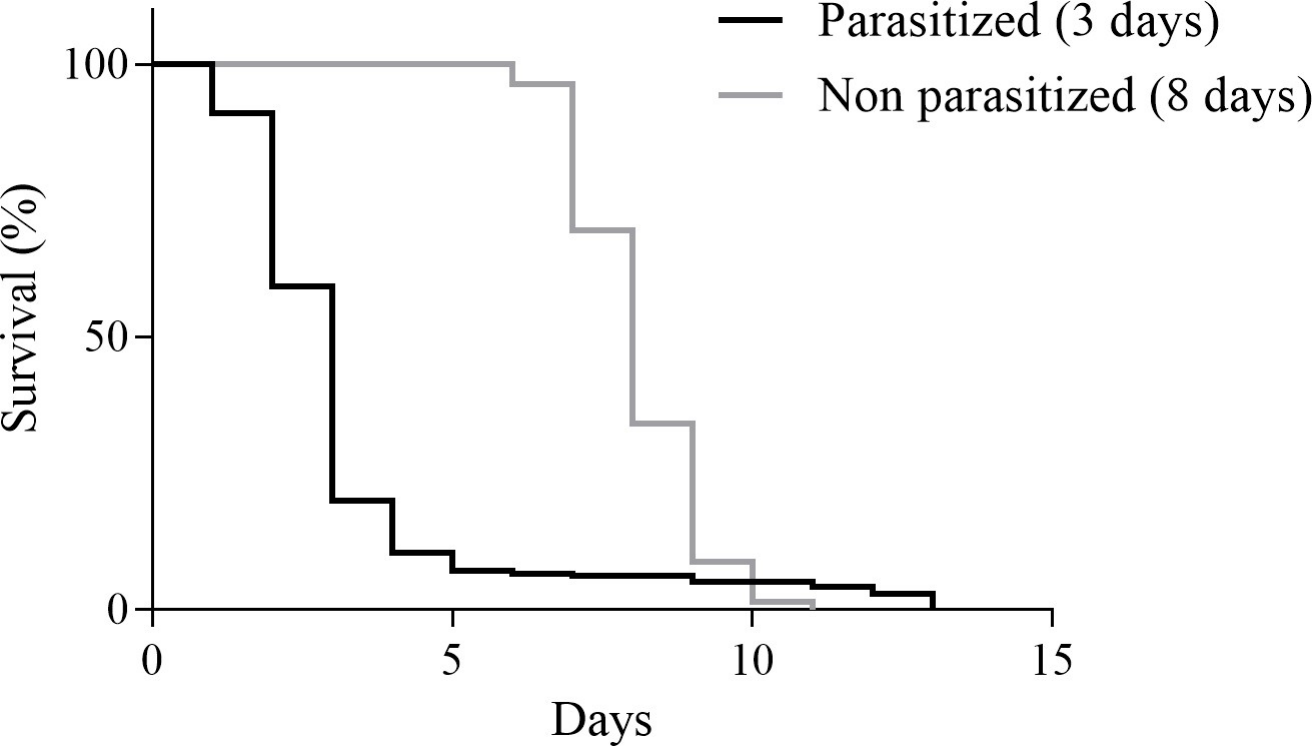
Percent survival of parasitized *Atta bisphaerica* workers and non-parasitized by *Apocephalus attophilus*. Median survival times (days) are shown in parenthesis.

**Fig 2:**
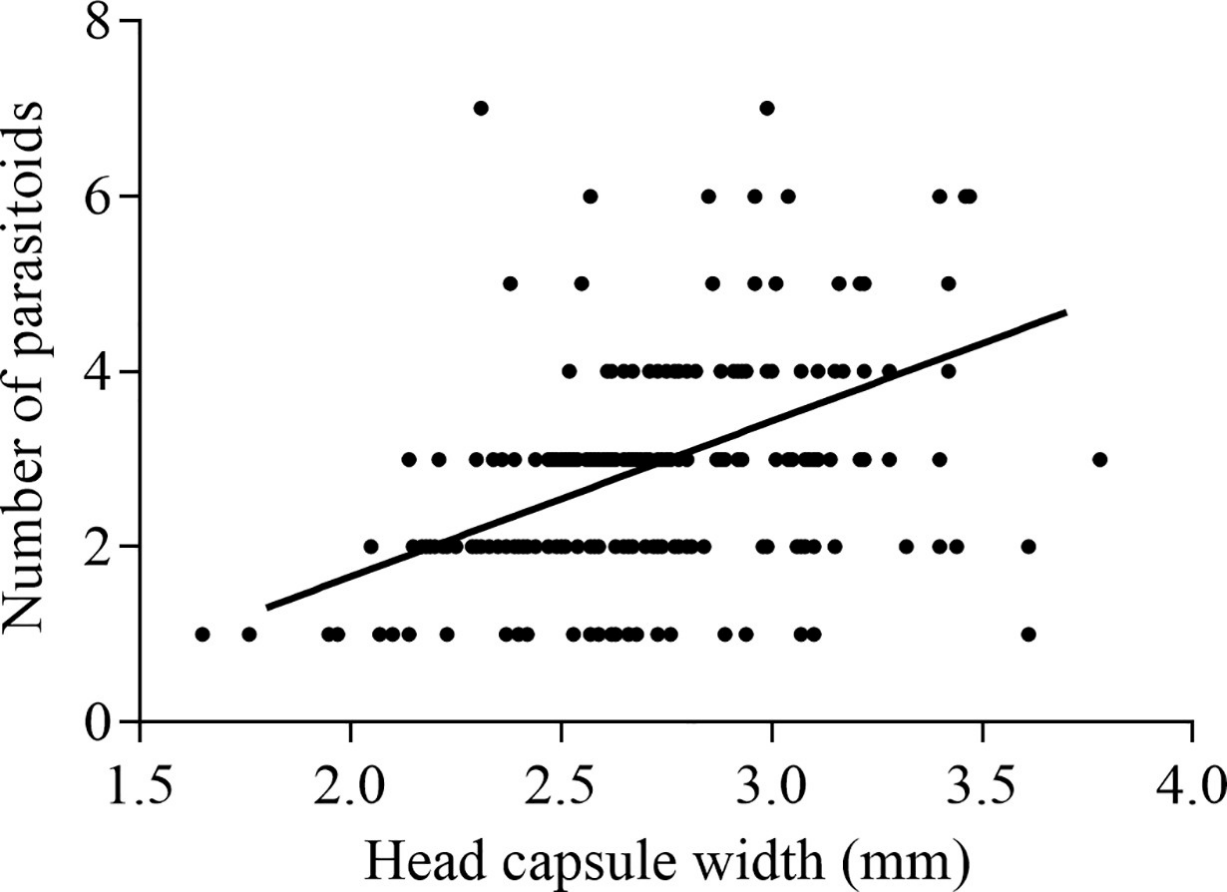
Correlation between the head size of parasitized *Atta bisphaerica* and the number of *Apocephalus attophilus* pupae (*r* = 0.40; *P* <0.0001; n = 211).

**Fig 3:**
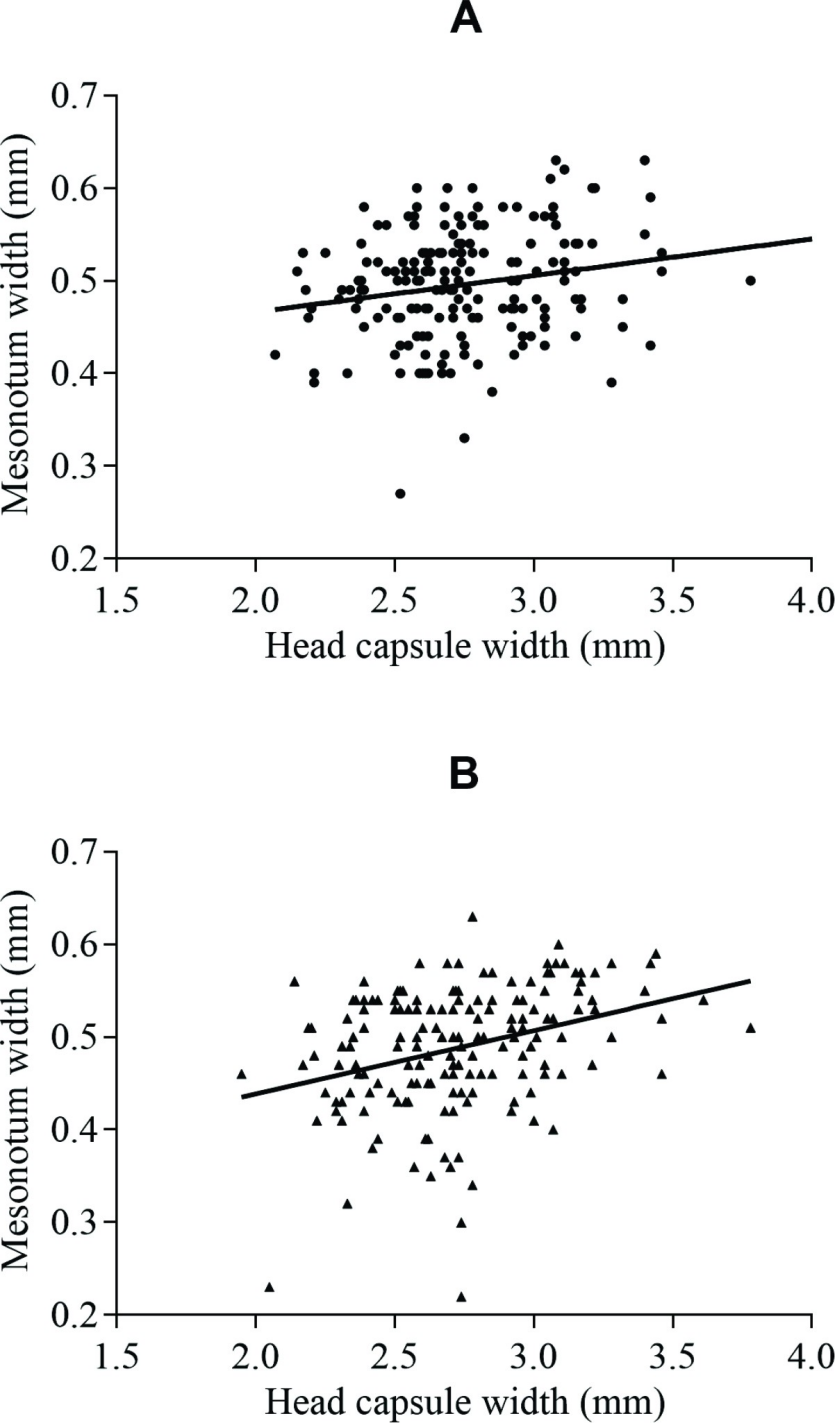
**Correlation between head size of parasitized *Atta bisphaerica* and size of (A) females** (*r* = 0.22; *P* = 0.002; n = 188) **and (B) males** (*r* = 0.33; *P*< 0.0001; n = 173) **of emerging *Apocephalus attophilus***.

When survival of non-parasitized ants by *A*. *attophilus* was compared with that of parasitized, we found a statistically significant difference between the survival curves. Non-parasitized ants (8.1 ± 0.1; n = 217) survived longer (*χ*^*2*^ = 204.4; df = 1; *P*< 0.0001) than those parasitized (3.2 ± 0.2; n = 217) (Fig 1). The number of *A*. *attophilus* pupae per host increased with the host head size (*r* = 0.40; *P*< 0.0001; n = 211) (Fig 2). Likewise, the size of the adult parasitoids female (*r* = 0.22; *P* = 0.002; n = 188) and male (*r* = 0.33; *P*< 0.0001; n = 173) also increased depending on the host ant (Fig 3).

The larval and pupal periods of *A*. *attophilus* were 2.2 ± 0.8 (n = 217) and 16 ± 1.4 days (n = 169), respectively. The number of pupae of this parasitoid per parasitized ant ranged from 1 to 7 (2.6 ± 1.3 pupae/ant) and were found externally on the ants body. The percentage of parasitoid emerging adults was 77.8.

Differences in wing length showed that *A*. *attophilus* females (1.5 ± 0.1 mm; n = 166) were larger (*t* = 7.63; *P*< 0.0001) than males (1.3 ± 0.2 mm; n = 152) and the sex ratio (male: female) did not differ from 1:1 (*χ*^*2*^ = 0.63; df = 1; *P* = 0.42; n males = 192 and n females = 209).

## Discussion

The parasitism rate of *A*. *bisphaerica* workers by phorids observed in this study (0.99%) was below 6%, similar to that reported for *A*. *bisphaerica* (4.37%) [18], *A*. *laevigata* (2.8 and 5.36%) [36,37], *A*. *sexdens* (1.57 and 2.94%) [37,31], *Atta vollenweideri* (Forel) (0.08 to 3.88%) [8] and *Solenopsis* spp. (0.2 to 2.4%) [35,38] by their respective phorid parasitoids. Differences in parasitism rates may be related to the climatic variables such as temperature and precipitation in the different studied sites that affect the phenology of the phorids [8,39]. Although reported parasitism rates were low, impacts on normal colony activities caused by these parasitoids suggest that these insects have potential for use in biological control programs of ant pests [8,11,12].

The highest parasitism rate by *A*. *attophilus* in the hottest/rainy season was different from reported for this parasitoid in other studies that found a higher parasitism in colder (Farder-Gomes et al. 2018 unpublished work) and drier seasons [18], suggesting that *A*. *attophilus* has a remarkable physiological plasticity, being able to parasitize ants during hot and rainy seasons.

The survival of workers parasitized by *A*. *attophilus* was lower than that of non-parasitized ants, because the larvae of the parasitoid consume the cephalic content of the host ant, killing it before pupating [23]. The survival of hosts parasitized by *A*. *attophilus* is lower than the survival of hosts parasitized by other phorid flies [18,31,36,37]. In addition, the pupal period of *A*. *attophilus* found in this study was similar to that already reported for this parasitoid and also lower than that of other phorids studied [18,23,31,36,37]. As this parasitoid kills the ants faster, reducing the damage caused by these pests, it can be considered a potential biocontrol agent.

The positive correlation between host size and the number and size of emerging *A*. *attophilus* may be due to the greater amount of resources available to the development of more larvae and larger parasitoids [27,31]. Similarly, the number of *A*. *attophilus* pupae per host increases with the head size of *A*. *bisphaerica* [18], *A*. *laevigata* [23] and *A*. *sexdens* [31]. Larger phorids of *E*. *bragancai* and *M*. *grandicornis* occur in larger workers of *A*. *bisphaerica* and *A*. *sexdens*, respectively [18,19]. Therefore, in order to produce parasitoids under laboratory conditions to control leaf-cutting ants, bigger ant workers are more suitable, because in these hosts, flies with a larger body size and in higher numbers are produced.

The number of *A*. *attophilus* pupae per host ranged from 1 to 7 (2.64 ± 1.30 pupae/ant), whereas other studies with *A*. *bisphaerica*, *A*. *sexdens* and *A*. *laevigata* reported variations from 1 to 10 (2.50 ± 1.12), 1 to 16 and 4 to 7 pupae/ant, respectively [18,31,36]. This finding together with observed correlation between host size and number of parasitoids/host, indicates that *A*. *attophilus* is a gregarious parasitoid in which several offspring successfully develop on each host similar to that reported for the same species [18,23,31,36] and gregarious hymenopteran parasitoids [40–42].

*Apocephalus attophilus* females are larger than males and this is likely due to the higher costs related to the production, maturation and oviposition of the eggs by females and to the greater accumulation of nutritional reserves [27,43]. The greater body size of females compared to males has already been reported for the parasitoids of leaf-cutting ants *Eibesfeldtphora elongata* (Brown) [28], *M*. *grandicornis* [19] and *Eibesfeldtphora tonhascai* (Brown) [44]. Individuals with larger bodies have more resources to allocate in reproductive parameters, such as number of ovarioles, egg production, longevity, fecundity and life span [29,42,45–47]. In addition, larger *A*. *attophilus* females may have a higher capacity to sustain longer periods of flight, higher host search ability and ability to sustain longer oviposition bouts on ants [27]. Several studies have shown that females gain more than males in fitness by being larger [27,48–51].

The 1: 1 sex ratio in *A*. *attophilus* corroborates with the results observed for other species of phorid flies attacking leaf-cutting ants [8,9,31]. Sex ratio variation as a function of host size occurs in phorids of the genus *Pseudacteon*, in which females commonly emerge from larger hosts than males, suggesting that the amount of food ingested by larvae is a possible sex determination mechanism in these parasitoids [26,27]. However, Farder-Gomes et al. (2016) found no difference between the head capsule width of parasitized ants from which males and females emerged. It is possible that other factors, such as temperature and/or genetics, can influence the sex determination of phorid parasitoids of leaf-cutting ants, similar to that reported for *Megaselia scalaris* (Loew) and *Megaselia halterata* (Wood) [25,52].

Our results indicate that *A*. *attophilus* is a gregarious parasitoid that parasitized *A*. *bisphaerica* in hot and rainy periods. Parasitized ants by this species survived significantly less than non-parasitized ones. The host size affects the number and size of the offspring emerged and parasitoid females are larger than males. Additionally, our results can be important for those interested in massive rearing program of phorid parasitoids for biological control purposes. *Apocephalus attophilus* is a potential biocontrol agent of leaf-cutting ants, mainly *A*. *bisphaerica*, because this parasitoid produces multiple larvae per host, allowing a great production of parasitoids with short developmental time and kills the host ant faster than other phorids, reducing the damage caused by leaf-cutting ants.

## References

[1] Della Lucia TM, Gandra LC, Guedes RNC. Managing leaf-cutting ants: Peculiarities, trends and challenges. Pest Manag Sci. 2014;70: 14–23. 10.1002/ps.3660 24115496 10.1002/ps.3660

[2] Fowler HG, Pagani MI, Da Silva OA, Forti LC, Da Silva VP, De Vasconcelos HL. A pest is a pest is a pest? The dilemma of neotropical leaf-cutting ants: Keystone taxa of natural ecosystems. Environ Manage. 1989;13: 671–675. 10.1007/BF01868306

[3] Della Lucia TM. *Atta bisphaerica*: uma ilustre desconhecida. Naturalia. 1999;24: 53–59.

[4] AgroSciences D. Controle de formigas-cortadeiras. 1998.

[5] Damalas CA, Eleftherohorinos IG. Pesticide exposure, safety issues, and risk assessment indicators. Int J Environ Res Public Health. 2011;8: 1402–1419. 10.3390/ijerph8051402 21655127 10.3390/ijerph8051402 PMC3108117

[6] Guillade AC, Folgarait PJ. Natural Enemies of *Atta vollenweideri* (Hymenoptera: Formicidae) Leaf-Cutter Ants Negatively Affected by Synthetic Pesticides, Chlorpyrifos and Fipronil. J Econ Entomol. 2014;107: 105–114. 10.1603/EC12498 24665691 10.1603/ec12498

[7] Han W, Tian Y, Shen X. Human exposure to neonicotinoid insecticides and the evaluation of their potential toxicity: An overview. Chemosphere. 2018;192: 59–65. 10.1016/j.chemosphere.2017.10.149 29100122 10.1016/j.chemosphere.2017.10.149

[8] Guillade AC, Folgarait PJ. Life-History Traits and Parasitism Rates of Four Phorid Species (Diptera: Phoridae), Parasitoids of *Atta vollenweideri* (Hymenoptera: Formicidae) in Argentina. J Econ Entomol. 2011;104: 32–40. 10.1603/EC10173 21404836 10.1603/ec10173

[9] Folgarait PJ. Leaf-cutter ant parasitoids: Current knowledge. Psyche. 2013;2013 10.1155/2013/539780

[10] Tonhasca A. Jr Interactions Betwen a Parasitic Fly, *Neodohrniphora declinata* (Diptera: Phoridae), and its host, the leaf-cutting ant *Atta sexdens rubropolosa* (Hymenoptera: Formicidae). Ecotropica. 1996;2: 157–164.

[11] Bragança MAL, Tonhasca A Jr, Della Lucia TMC. Reduction in the foraging activity of the leaf-cutting ant *Atta sexdens* caused by the phorid *Neodohrniphora* sp. Entomol Exp Appl. 1998;89: 305–311.

[12] Guillade AC, Folgarait PJ. Effect of phorid fly density on the foraging of *Atta vollenweideri* leafcutter ants in the field. Entomol Exp Appl. 2015;154: 53–61. 10.1111/eea.12255

[13] Graham LCF, Porter SD, Pereira RM, Dorough HD, Kelley AT. Field releases of the Decapitating Fly *Pseudacteon curvatus* (Diptera: Phoridae) for Control of Imported Fire Ants (Hymenoptera: Formicidae) in Alabama, Florida, and Tennessee. Florida Entomol. 2003;86: 334–339. 10.1653/0015-4040(2003)086[0334:FROTDF]2.0.CO;2

[14] Callcott AMA, Porter SD, Weeks RD Jr, Graham LCF, Johnson SJ, Gilbert LE. Fire ant decapitating fly cooperative release programs (1994–2008): Two *Pseudacteon* species, *P*. *tricuspis* and *P*. *curvatus*, rapidly expand across imported fire ant populations in the southeastern United States. J Insect Sci. 2011;11:1–25. 10.1673/031.011.0101 or http://www.insectscience.org/11.19/21526930 10.1673/031.011.0119 PMC3281391

[15] Porter SD, Graham LCF, Johnson SJ, Thead LG, Briano JA. The large decapitating fly *Pseudacteon litoralis* (Diptera: Phoridae) successfully established on fire ant populations in Alabama. Florida Entomol. 2011;94: 208–213. http://www.fcla.edu/FlaEnt/ or 10.1653/024.094.0213

[16] Orr MR. Parasitic flies (Diptera: Phoridae) influence foraging rhythms and caste division of labor in the leaf-cutter ant, *Atta cephalotes* (Hymenoptera: Formicidae). Behav Ecol Sociobiol. 1992;30: 395–402.

[17] Bragança MAL, Della Lucia TMC, Tonhasca A Jr. First record of phorid parasitoids (Diptera: Phoridae) of the leaf-cutting ant *Atta bisphaerica* Forel (Hymenoptera: Formicidae). Neotrop Entomol. 2003;32: 169–171. 10.1590/S1519-566X2003000100028

[18] Martins HC. Aspectos da biologia e ecologia de três espécies de forídeos parasitoides da saúva Atta bisphaerica. M. Sc. Thesis, Universidade Federal de Viçosa. 2015. Available from: http://www.locus.ufv.br/handle/123456789/7266

[19] Tonhasca A Jr, Bragança MAL, Erthal M Jr. Parasitism and biology of *Myrmosicarius grandicornis* (Diptera, Phoridae) in relationship to its host, the leaf-cutting ant *Atta sexdens* (Hymenoptera: Formicidae). Insectes Soc. 2001;48: 154–158. 10.1007/PL00001759

[20] Bragança MAL, Tonhasca A Jr, Moreira DDO. Parasitism characteristics of two phorid fly species in relation to their host, the leaf-cutting ant *Atta laevigata* (Smith) (Hymenoptera: Formicidae). Neotrop Entomol. 2002;31: 241–244. 10.1590/S1519-566X2002000200010

[21] Feener DH Jr, Moss KAG. Defense against parasites by hitchhikers in leaf-cutting ants: a quantitative assessment. Behav Ecol Sociobiol. 1990;26: 17–29. 10.1007/BF00174021

[22] Brown BV. Revision of the *Apocephalus attophilus* group of ant-decapitating flies (Diptera: Phoridae). Contrib Sci. 1997;468: 1–60. 10.11646/zootaxa.3857.4.5 25283122

[23] Erthal M Jr, Tonhaasca A Jr. Biology and oviposition behavior of the phorid *Apocephalus atoaphilus* and the response of its host, the leaf-cutting ant *Atta laevigata*. Ent Exp Appl. 2000;95: 71–75.

[24] Morrison LW, Gilbert LE. Parasitoid-host relationships when host size varies: The case of *Pseudacteon* flies and *Solenopsis* fire ants. Ecol Entomol. 1998;23: 409–416. 10.1046/j.1365-2311.1998.00159.x

[25] Disney RHL. Natural History of the Scuttle Fly, *Megaselia scalaris*. Annu Rev Entomol. 2008;53: 39–60. 10.1146/annurev.ento.53.103106.093415 17622197 10.1146/annurev.ento.53.103106.093415

[26] Chirino MG, Folgarait PJ, Gilbert LE. *Pseudacteon tricuspis*: Its Behavior and Development According to the Social Form of Its Host and the Role of Interference Competition Among Females. J Econ Entomol. 2012;105: 386–394. 10.1603/EC09170 22606808 10.1603/ec09170

[27] Morrison LW, Porter SD, Gilbert LE. Sex ratio variation as a function of host size in *Pseudacteon* flies (Diptera: Phoridae), parasitoids of *Solenopsis* fire ants (Hymenoptera: Formicidae). Biol J Linn Soc. 1999;66: 257–267.

[28] Bragança MAL, Tonhasca A Jr, Della Lucia TMC. Características biológicas e comportamentais de *Neodohrniphora elongata* Brown (Diptera, Phoridae), um parasitóide da saúva *Atta sexdens rubropilosa* Forel (Hymenoptera, Formicidae). Rev Bras Entomol. 2009;53: 600–606. 10.1590/S0085-56262009000400009

[29] Jervis MA, Ellers J, Harvey JA. Resource Acquisition, Allocation, and Utilization in Parasitoid Reproductive Strategies. Annu Rev Entomol. 2008;53: 361–385. 10.1146/annurev.ento.53.103106.093433 17877453 10.1146/annurev.ento.53.103106.093433

[30] Gao S, Tang Y, Wei K, Wang X, Yang Z, Zhang Y. Relationships between body size and parasitic fitness and offspring performance of *Sclerodermus pupariae* Yang et Yao (Hymenoptera: Bethylidae). PLoS One. 2016;11: e0156831 10.1371/journal.pone.0156831 27367523 10.1371/journal.pone.0156831 PMC4930212

[31] Farder-Gomes CF, Oliveira MA, Gonçalves PL, Gontijo LM, Zanuncio JC, Bragança MAL, et al Reproductive ecology of phorid parasitoids in relation to the head size of leaf-cutting ants *Atta sexdens* Forel. Bull Entomol Res. 2016;107: 487–492. 10.1017/S0007485316001073 27903323 10.1017/S0007485316001073

[32] Brown BV. Taxonomic revision of *Neodohrniphora*, subgenus *Eibesfeldtphora* (Diptera: Phoridae). Insect Syst Evol. 2001;32: 393–409.

[33] Disney RHL, Elizalde L, Folgarait PJ. New species and revision of *Myrmosicarius* (Diptera: Phoridae) that parasitize leaf-cutter ants (Hymenoptera: Formicidae). Sociobiology. 2006;47: 771–809.

[34] Uribe S, Brown BV, Braganca MAL, Queiroz JM, Nogueira CA. New species of *Eibesfeldtphora* Disney (Diptera: Phoridae) and a new key to the genus. Zootaxa. 2014;3814: 443–50. doi: 10.11646/zootaxa.3814.3.11 24943441 10.11646/zootaxa.3814.3.11

[35] Morrison LW, Dall’Aglio-Holvorcem CG, Gilbert LE. Oviposition behavior and development of *Pseudacteon* flies (Diptera: Phoridae), parasitoids of *Solenopsis* fire ants (Hymenoptera: Formicidae). Environ Entomol. 1997;26: 716–724.

[36] Bragança MAL, Medeiros ZCS. Ocorrência e características biológicas de forídeos parasitóides (Diptera: Phoridae) da saúva *Atta laevigata* (Smith) (Hymenoptera: Formicidae) em Porto Nacional, TO. Neotrop Entomol. 2006;35: 408–411. 10.1590/S1519-566X2006000300018 18575704 10.1590/s1519-566x2006000300018

[37] Bragança MAL, Arruda FV, Souza LRR, Martins HC, Della Lucia TMC. Phorid Flies Parasitizing Leaf-Cutting Ants: Their Occurrence, Parasitism Rates, Biology and the First Account of Multiparasitism. Sociobiology. 2016;63: 1015–1021. doi: 10.13102/sociobiology.v63i4.1077

[38] Calcaterra LA, Delgado A, Tsutsui ND. Activity patterns and parasitism rates of fire ant-decapitating flies (Diptera: Phoridae: *Pseudacteon* spp.) in their native Argentina. Ann Entomol Soc Am. 2008;101: 539–550.

[39] Folgarait PJ, Bruzzone OA, Gilbert LE. Seasonal patterns of activity among species of black fire ant parasitoid flies (*Pseudacteon*: Phoridae) in Argentina explained by analysis of climatic variables. Biol Control. 2003;28: 368–378. 10.1016/S1049-9644(03)00093-8

[40] Charnov EL, Skinner SW. Evolution of host selection and clutch size in parasitoid wasps. Fla Entomol. 1984;67: 5–21. 10.2307/3494101

[41] Bell HA, Marris GC, Prickett AJ, Edwards JP. Influence of host size on the clutch size and developmental success of the gregarious ectoparasitoid *Eulophus pennicornis* (Nees) (Hymenoptera: Braconidae) attacking larvae of the tomato moth *Lacanobia oleracea* (L.) (Lepidoptera: Noctuidae). J Exp Biol. 2005;208: 3199–3209. 10.1242/jeb.01759 16081616 10.1242/jeb.01759

[42] Liu Z, Xu B, Li L, Sun J. Host-size mediated Trade-Off in a Parasitoid *Sclerodermus harmandi*. PLoS One. 2011;6: e23260 10.1371/journal.pone.0023260 21853096 10.1371/journal.pone.0023260 PMC3154928

[43] Foo F-K, Othman AD, Lee C-Y. Effects of Body Size on the Biological Fitness of a Koinobiotic Phorid Parasitoid and on the Parasitoid–Termite Host Relationship. Ann Entomol Soc Am. 2016;110: 227–232. 10.1093/aesa/saw078

[44] Bragança MAL, Oliveira RJ, Della Lucia TMC. Influência do tamanho das operárias de *Atta sexdens rubropilosa* (Hymenoptera: Formicidae) sobre a razão sexual e tamanho do parasitóide *Neodohrniphora tonhascai* (Diptera: Phoridae). Biol. 2007;69: 447–449.

[45] Arakawa R, Miura M, Fujita M. Effects of host species on the body size, fecundity, and longevity of *Trissolcus mitsukurii* (Hymenoptera: Scelionidae), a solitary egg parasitoid of stink bugs. Appl Entomol Zool. 2004;39: 177–181. 10.1303/aez.2004.177

[46] Saeki Y, Crowley PH. The size-number trade-off and components of fitness in clonal parasitoid broods. Entomol Exp Appl. 2013;149: 241–249. 10.1111/eea.12130

[47] Segoli M, Rosenheim JA. The effect of body size on oviposition success of a minute parasitoid in nature. Ecol Entomol. 2015;40: 483–485. 10.1111/een.12194

[48] Charnov EL, Los-den Hartogh RL, Jones WT, van den Assem J. Sex ratio evolution in a variable environment. Nature. 1981;289: 27–33. 7453809 10.1038/289027a0

[49] Hurlbutt B. Sexual size dimorphism in parasitoid wasps. Biol J Linn SocieQ. 1987;30: 63–89.

[50] Ueno T. Host-size-dependent sex ratio in a parasitoid wasp. Res Popul Ecol. 1999;41: 47–57. 10.1007/PL00011982

[51] Cloutier C, Duperron J, Tertuliano M, McNeil JN. Host instar, body size and fitness in the koinobiotic parasitoid *Aphidius nigripes*. Entomol Exp Appl. 2000;97: 29–40. 10.1023/A:1004056818645

[52] Barzegar S, Zamani AA, Abbasi S, Vafaei Shooshtari R, Shirvani Farsani N. Temperature-Dependent Development Modeling of the Phorid Fly *Megaselia halterata* (Wood) (Diptera: Phoridae). Neotrop Entomol. 2016;45: 507–517. 10.1007/s13744-016-0400-3 27147228 10.1007/s13744-016-0400-3

